# Preoperative clinical predictors of lymph node metastasis in endometrial cancer: a retrospective cohort study

**DOI:** 10.1186/s12905-025-04249-2

**Published:** 2026-01-22

**Authors:** Ahmed Metwally Elkattawy, Mohamed Mahmoud Almeniawy, Mohamed Adel Saleh, Alaa Haggag, Ahmed Elsayed Mansor, Ola A. Harb, Loay M. Gertallah, Amany M. Abdallah, Mahmood Ahmed Osman, Mustafa Omara

**Affiliations:** 1 Faculty of Medicine, Zagazig University, Zagazig, Egypt; 2 Zagazig University Hospitals, Zagazig University, Zagazig, Egypt

**Keywords:** Endometrial carcinoma, Lymph node metastases, Predictive parameters

## Abstract

**Background:**

Many low-risk endometrial cancer (EC) patients will get no significant benefits from unneeded lymphadenectomy in addition to increasing morbidity and lymphedema risks, which worsen patients’ prognosis. In the present study, we aimed to develop a non-invasive model for the detection of lymph node metastases in endometrial cancer patients using relatively simple clinical predictors.

**Patients and methods:**

We included a total of 100 EC patients. We collect the demographic parameters, serologic markers, imaging parameters, and FIGO pathologic staging. Preoperative pathological assessment of the endometrial biopsy was evaluated. We considered grades 1 and 2 endometrioid EC as low-grade endometrial carcinoma (LGEC), and another histological grade as high-grade endometrial carcinoma (HGEC). We correlate between patients with and without lymph node metastases regarding all detected parameters.

**Results:**

Of the 100 patients, 53 had lymph node metastases, and 47 had no lymph node metastases. Radiological evidence of enlarged lymph nodes, large tumor volume, lympho-vascular invasion (LVI), and high levels of serum CA125 (U/mL) were positively associated with the presence of lymph node metastases (*p* < 0.001). Patients with pathological evidence of lymph node metastases, who were predicted to have lymph node metastases preoperatively based on clinical and radiological findings, demonstrated less favorable prognostic outcomes compared to those with negative models of prediction.

**Conclusions:**

High serum CA125, enlarged lymph nodes, and large tumor volume were positively associated with the presence of lymph node metastases. While other factors, such as deep myometrial invasion, deep cervical stromal invasion, showed significant associations. This predictive model may assist clinicians in identifying high-risk patients who truly benefit from lymphadenectomy, while sparing low-risk patients from unnecessary procedures and their associated complications.

## Introduction

Endometrial cancer (EC) is the fourth most common cancer in females and the most common gynecologic cancer in developed countries [1]. Recently, most EC cases have been detected in the early stage, and their associated mortality rate has become the lowest among gynecologic cancers [2]. The main prognostic parameter of the prognosis of EC patients is the presence or absence of lymph node metastasis, which is assessed surgically by lymph node biopsy or lymphadenectomy. Many low-risk endometrial cancer patients gain no significant benefit from lymphadenectomy, which may instead increase morbidity and the risk of lymphedema, ultimately worsening their prognosis [3]. Additionally, the therapeutic benefits of performing lymphadenectomy in EC patients are still under research [4]. Diagnosing the presence or absence of lymph node metastases in EC remains an important step in a patient’s prognosis [5]. Thus, non-invasive predictive parameters of lymph node metastases in EC patients allow adequate risk stratification, assess patients’ prognosis, make adequate decisions, and avoid unneeded lymphadenectomy.

Previous reports demonstrated that clinical factors such as the presence and degree of myometrial invasion and tumor morphology allow pre-operative prediction of lymph node metastases without performing lymphadenectomy. These reports led to the detection of many scoring systems [6, 7].

The Kanagawa Cancer Center (KCC) system was designed to assess lymphadenectomy extent in EC patients [6], and this system considered that factors associated with lymph node metastases in EC patients are any histopathological sub-type other than endometroid endometrial carcinoma, tumor volume (> 6 cm^3^), high level of CA125 level and presence of myometrial invasion more than 50% of thickness. Former scoring systems were simple and easily used in clinical practice, but they were not sufficiently validated by multi-institutional studies.

Most previous models have relied on either serologic markers, MRI findings, or pathological features individually, with very few integrating all three domains. In the present study, we aimed to develop and assess a non-invasive predictive model for detecting lymph node metastases in endometrial cancer patients by combining MRI findings, CA125 levels, and tumor size in both low- and high-risk EC patients.

## Patients and methods

In the present retrospective cohort study, all patients diagnosed with endometrial cancer (EC) and admitted to the Gynecology and Obstetrics Department or the General Surgery Department at Zagazig University Hospitals between 2017 and 2023 were included in the study. We included all patients diagnosed in our center; this cohort reflects both high-risk and low-risk populations for better evaluation of external validity. All patients underwent radical hysterectomy and bilateral salpingo-oophorectomy with a minimum of 10 lymph nodes removed during surgery. Extra-facial hysterectomy and radical hysterectomy were performed for stage 2 cases. Among the included patients, 15% underwent a laparoscopic surgical approach, while the remaining 85% underwent open surgery. Sentinel lymph node mapping was performed in 15% of cases. Exclusion criteria: patients with pre-operative neoadjuvant chemotherapy and patients who have not undergone lymphadenectomy were excluded. After applications of strict inclusion criteria, we included a total of 100 EC patients.

The following parameters were collected for all included patients: demographic parameters (age, body mass index (BMI), menopause, gravidity, parity, patient and family history of cancer breast and colon), serologic markers )CA 19 − 9, CA125, and CEA), imaging parameters (index of tumor volume, presence and degree of myometrial invasion, presence and degree of cervical stromal invasion, presence and laterality of adnexal invasion, enlarged pelvic and/or para-aortic lymph nodes and presence of distal metastasis) and FIGO pathologic staging.

Preoperative pathological assessment of the endometrial biopsy was evaluated. Endometrioid EC of grades 1 and 2 were classified as low-grade endometrial carcinoma (LGEC), while other histological grades were classified as high-grade endometrial carcinoma (HGEC). Large tumor diameter was defined as greater than 8 cm on MRI.

Patients were categorized into premenopausal and postmenopausal groups to assess the association between serum CA125 levels and pathological findings, given that CA125 levels are influenced by age and ovarian hormone levels. Serum CA125 (Cancer Antigen 125) levels were measured in units per milliliter (U/mL) and assessed as a potential predictor of lymph node metastasis.

### Statistical analysis

We performed statistical analysis using JMP version 15.0.0 software (SAS Institute Inc., Cary, NC, USA) and R software ver. 4.1.0 (R Foundation, Vienna, Austria). We used t-test and Chi-square tests according to the type of data. We subsequently included statistically significant variables in the univariate analysis in the multivariate analysis. The *p* < 0.05 is considered the level of statistical significance. We estimated cumulative survival rates by using the Kaplan–Meier curves, and correlated differences in survival rates between both included groups by using the log-rank test. Multivariate logistic regression analysis was performed, and cross-validation was used in the predictive model to ensure better internal validation and robustness of the results.

### Ethical approval

Ethical approval for the study was obtained from the Institutional Review Board of the Faculty of Medicine, Zagazig University (IRB No. 492/21-July-2024). Written informed consent was obtained from all participants.

## Results

Table 1 presents the baseline demographic and clinical characteristics of the 100 included EC patients.

**Table 1:** Baseline data of studied patients

	Total patients *N* = 100 (%)
Baseline data	
Age (year) [mean ± SD]	57.05 ± 9.69
BMI (kg/m2) [mean ± SD]	29.56 ± 5.19
History	
Gravidity [median (IQR)]	1(1–3)
Parity [median (IQR)]	1(0.25–3)
Menopause n (%)	85 (85%)
History of breast cancer n (%)	18 (18%)
History of cancer colon n (%)	9 (9%)
Serologic markers	
CEA [median (IQR)]	3.5(2.1–7.53)
CA 19 − 9 [median (IQR)]	175.5(66.3–537.5)
CA 125 (U/mL) [median (IQR)]	225(50–597.5)
MRI findings	
Myometrial invasion > 50% n (%)	52 (52%)
Cervical stromal invasion n (%)	37 (37%)
Distant metastasis n (%)	15 (15%)
Enlarged pelvic LN n (%)	27 (27%)
LVI n (%)	20 (20%)
Enlarged paraaortic LN n (%)	33 (33%)
Adnexal involvement n (%)	19 (19%)
Large tumor diameter n (%)	52 (52%)
Pathology	
Non-G1 n (%)	50 (50%)
Lymph node metastasis	53 (53%)

The mean age was 57.05 years, and the mean BMI was 29.56 kg/m². A history of breast cancer was reported in 18% of patients, while 9% had a history of colorectal cancer (CRC). MRI findings of patients revealed that 52% had myometrial invasion > 50%, 37%, and 15% had cervical stromal affection and distant metastasis, respectively, 27% and 33% had enlarged pelvic and paraaortic LN, respectively, and adnexal involvement was noted in 19% and 52% had large tumor volume. Pathology revealed that 50% had a grade > grade 1. Lymph node metastasis was found in 53% of patients.

### Associations between preoperative clinicopathological parameters and risks of lymph node metastases

We showed that radiological evidence (as detected by MRI) of deep myometrium invasion by the tumor, enlarged lymph nodes, large tumor volume, and deep cervical stromal invasion, in addition to high levels of serum CA125, were positively associated with the presence of lymph node metastases in the included EC patients (*p* < 0.001). A statistically significant difference was observed between patients with and without lymph node metastases regarding tumor grade, with non-G1 tumors being more frequent among patients with lymph node metastasis (*p* = 0.003). Moreover, lympho-vascular invasion (LVI) was strongly associated with lymph node metastases. LVI was defined as the presence of tumor emboli detected by histopathological analysis (*p* < 0.001) (Table 2).

**Table 2:** Relation between lymph node metastasis and the studied parameters

	Total patients *N* = 100 (%)	No LN metastasis *N* = 47 (%)	LN metastasis *n* = 53 (%)	Test	*p*
Baseline data					
Age (year)^§^	57.05 ± 9.69	55.62 ± 9.85	58.32 ± 9.45	-1.4	0.165
BMI (kg/m2) ^§^	29.56 ± 5.19	27.93 ± 5.19	31.0 ± 4.79	-3.084	0.003*
History					
Gravidity ^∞^	1(1–3)	1(1–3)	1(1–3)	-0.103	0.908
Parity^∞^	1(0.25–3)	1(0–2)	1(0.5–3)	-0.348	0.728
Menopause^¥^	85 (85%)	38 (44.7%)	47 (55.3%)	1.197	0.274
History of breast cancer^¥^	18 (18%)	11 (61.1%)	7 (38.9%)	1.755	0.185
History of cancer colon^¥^	9 (9%)	2 (22.2%)	7 (77.8%)	Fisher	0.167
Serologic markers					
CEA^∞^	3.5(2.1–7.53)	2.3(1.7–6.7)	3.6(2.2–7.8)	-2.437	0.015*
CA 19 − 9^∞^	175.5(66.3–537.5)	70(39–133)	340(200–630)	-5.631	< 0.001**
CA 125 (U/mL) ^∞^	225(50–597.5)	50(33–91)	450(300–700)	-6.665	< 0.001**
MRI findings^¥^					
Myometrial invasion > 50%	52 (52%)	19 (36.5%)	33 (63.5%)	4.76	0.029*
Cervical stromal invasion	37 (37%)	9 (24.3%)	28 (75.7%)	12.123	< 0.001**
Distant metastasis	15 (15%)	2 (13.3%)	13 (86.7%)	8.03	0.005*
Enlarged pelvic LN	27 (27%)	7 (25.9%)	20 (74.1%)	6.594	0.01*
LVI	20 (20%)	5 (5%)	15 (15%)	9.594	< 0.001**
Enlarged paraaortic LN	33 (33%)	7 (21.2%)	26 (78.8%)	13.149	< 0.001**
Adnexal involvement	19 (19%)	7 (36.8%)	12 (63.2%)	0.972	0.324
Large tumor diameter	52 (52%)	18 (34.6%)	34 (65.4%)	6.67	0.01*
Pathology^¥^					
Non-G1	50 (50%)	16 (32%)	34 (68%)	9.033	0.003*

^§^data is represented as mean ± SD and compared using an independent sample t-test

^∞^Data is represented as median (IQR) and compared using the Mann-Whitney test

^¥^Data is represented as a number (%) and compared using the chi-square test

*p < 0.05 is statistically significant

**p ≤ 0.001 is statistically highly significant

Large tumor diameter > 8 cm

There is a significant relationship between lymph node metastasis and all of BMI (*p* = 0.003), Ca-125, Ca 19.-9 (*p* < 0.001), CEA (*p* = 0.015), MRI findings of myometrial invasion > 50% (*p* = 0.029), cervical stromal invasion, enlarged pelvic and paraaortic lymph node (*p* < 0.001), large tumor volume (*p* = 0.01), distant metastasis (*p* = 0.005), and grade of tumor (*p* = 0.003).

**Fig. 1:**
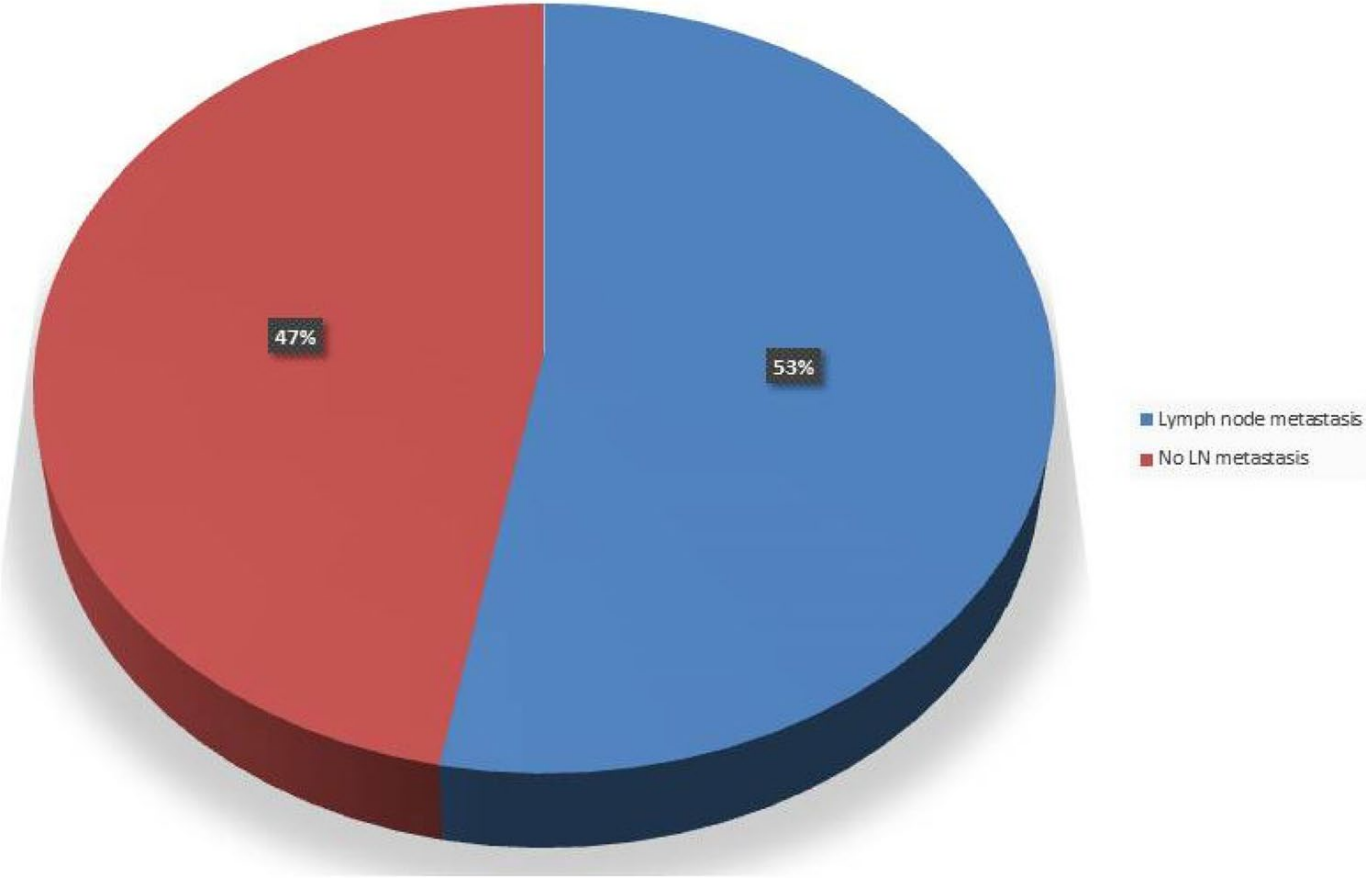
Proportional Distribution of LN metastasis Among EC Patients (*N* = 100). This pie chart shows the proportion of patients with and without LN metastasis confirmed by final pathological examination of dissected lymph nodes. LN metastasis was present in 53 patients (53%) and absent in 47 patients (47%)

These findings are presented in Table 2, and a visual representation is provided in Fig. 1.

### Predictive models and regression analysis for lymph node metastasis

Univariate analysis of predictors for lymph node metastasis revealed that increasing BMI, CA-125, and CA 19 − 9 significantly increase the risk of LN metastasis by 1.13, 1.007, and 1.004 folds, respectively. MRI findings of myometrial invasion > 50%, cervical stromal invasion (*p* < 0.001), distant metastasis, enlarged pelvic and paraaortic lymph node, and large tumor diameter significantly increase risk by 2.432, 4729, 3.463, 5.503, and 2.883 folds, respectively. Pathological findings of a grade higher than G1 significantly increased the risk by 3.467.

All significant factors in univariate analysis were included in backward Wald regression analysis to detect independent predictors for lymph node metastasis and the suggested model revealed an AUC (Area Under the ROC Curve) of 0.927, which reflects the model’s predictive accuracy and showed that higher CA-125, MRI findings of enlarged paraaortic LN, and large tumor diameter significantly independently increase risk by 1.007, 7.002 and 3.753 folds respectively. BMI non-significantly independently increases risk by 1.121-fold.

When introducing only MRI parameters, the AUC for the model was 0.807; when it included only significant serologic markers, it was 0.887, so the first model was the most acceptable.

**Fig. 2:**
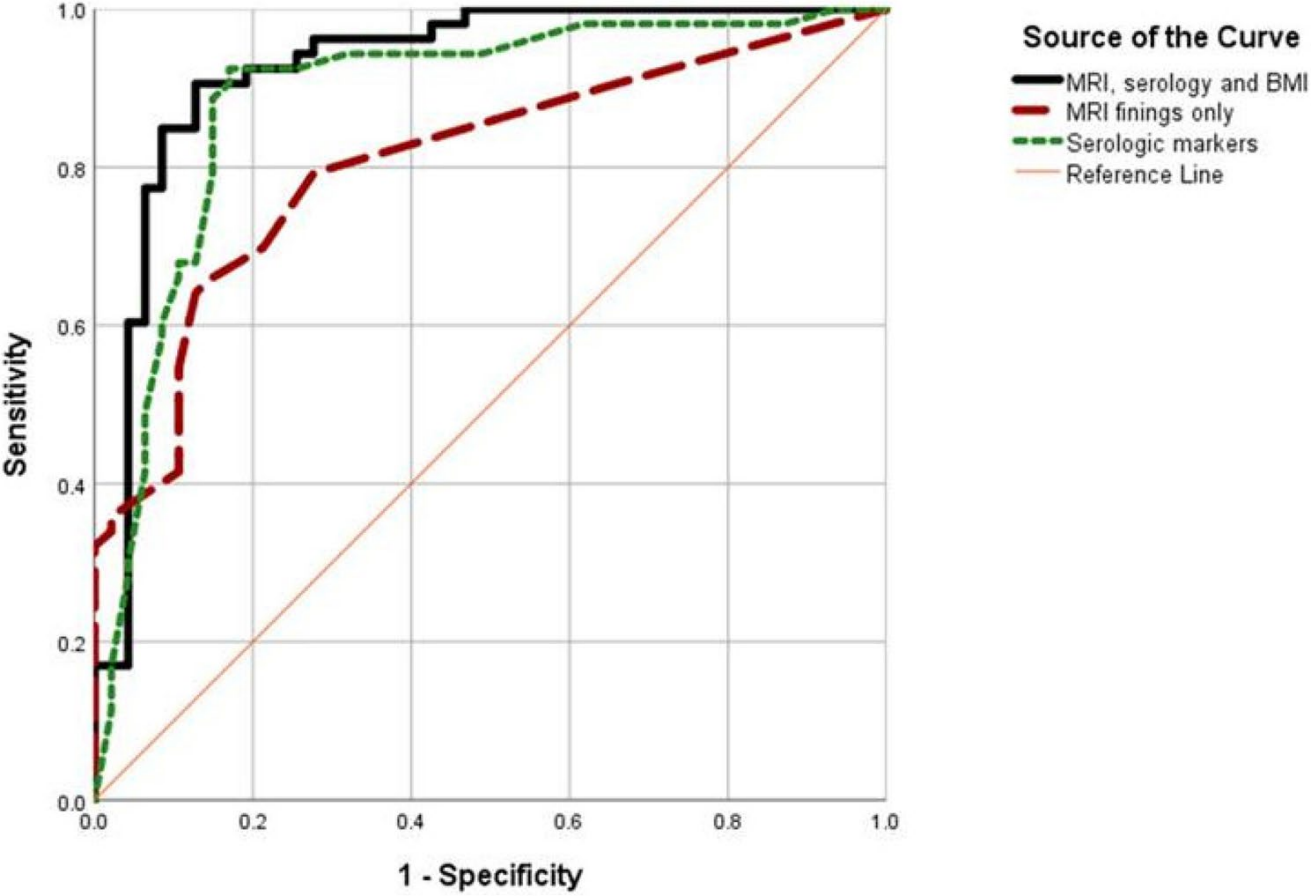
ROC curve for regression model of predictors of LN metastasis (AUC = 0.927 for model included MRI, serology, and BMI, AUC for MRI only was 0.807, while that for serology only was 0.887, *p* < 0.001)

The performance of the predictive models is detailed in Table 3, and a visual representation of the ROC curves is provided in Fig. 2.

**Table 3:** Univariate and multivariate analysis of factors associated with lymph node metastasis

	Univariate			Multivariate		
	COR	95% CI	*p*	AOR	95% CI	*p*
Age (year)	1.03	0.99–1.07	0.165			
BMI (kg/m2)	1.13	1.04–1.23	0.004*	1.121	0.993–1.266	0.065
Gravidity	1.017	0.78–1.33	0.902			
Parity	1.04	0.79–1.37	0.765			
Menopause	1.855	0.607–5.68	0.279			
History of breast cancer	0.498	0.18–1.41	0.19			
History of cervical cancer	3.424	0.68–17.38	0.138			
Non-G1	3.467	1.52–7.91	0.003*			
CEA	1.119	0.98–1.28	0.102			
CA 19 − 9	1.004	1–1.01	< 0.001**			
CA 125 (U/mL)	1.007	1.0–1.01	< 0.001**	1.007	1–1.01	< 0.001**
Myometrial invasion > 50%	2.432	1.09–5.44	0.03*			
Cervical stromal invasion	4.729	1.91–11.69	0.001**			
Distant metastasis	7.312	1.56–34.4	0.012*			
Enlarged pelvic LN	3.463	1.31–9.19	0.013*			
Enlarged paraaortic LN	5.503	2.09–14.47	0.001**	7.002	1.74–28.15	< 0.001**
Adnexal involvement	0.598	0.21–1.67	0.327			
Large tumor diameter	2.883	1.28–6.5	0.011*	3.753	1.11–12.7	0.033*

Large tumor diameter > 8 cm

*COR* crude odds ratio, *AOR* adjusted odds ratio, *CI* Confidence interval

*p < 0.05 is statistically significant

**p ≤ 0.001 is statistically highly significant

### Associations between clinical outcome and survival rates of the included patients and prediction of lymph node metastases

Patients with pathological evidence of lymph node metastases who had positive clinical and radiological preoperative prediction demonstrated worse outcomes compared to those with a negative prediction. No statistically significant differences in recurrence or survival rates were observed among patients without pathological evidence of lymph node metastases.

## Discussion

Discovering non-invasive predictive parameters of lymph node metastases in patients with EC leads to decreasing patients’ morbidity from unneeded lymphadenectomy. In the present study, we showed that deep myometrium invasion of more than 50%, deep cervical stromal invasion, lympho-vascular invasion, high serum CA125, and large tumor volume are associated with the high predictive power of the presence of lymph node metastases.

Our results were similar to the results of previous studies [8–12]. Additionally, Ueno et al. [8] and Asami et al. [9] showed that the group of patients with a negative predictive model and negative post-operative pathological evidence of lymph node metastases has better outcomes than the group of patients with a positive predictive model and positive post-operative pathological evidence of lymph node metastases.

Even patients with positive post-operative pathological evidence of lymph node metastases but a negative predictive model have better outcomes than patients with a positive predictive model Collectively these results in addition to our findings demonstrated that such a prediction model could identify patients at increased risks of tumor recurrence and unfavorable outcome, suggesting that such patients may benefit from postoperative therapy, even in the absence of pathological evidence of lymph node metastases after surgery.

Previous reports have performed a predictive model of lymph node metastases mainly in low-risk patients, but in the present study, we widened the study to include both high and low-risk patients. The presented predictive model has a high specificity, which allows an accurate prediction of the prognoses of patients who need lymphadenectomy.

Reijnen et al. [13] focused on the predictive value of the size of lymph nodes as detected by MRI and high levels of serum CA125 and showed that they are positively associated with a high incidence of lymph node metastases. It was found that clinical predictive factors of the presence of lymph node metastases in EC were associated with poor prognosis, so this model may help identify patients with poor prognosis, consistent with the findings of Asami et al. [9].

Many previous guidelines demonstrated that postoperative pathological evaluation of stage, histopathological subtype, grade, cervical stromal invasion, and lymph vascular invasion are considered predictive parameters for the assessment of prognosis and further management of EC patients [14, 15]. However, it would be more beneficial to detect pre-operative risk assessment and predictive parameters of EC patients to put the plan of management. While our study focuses on endometrial cancer, it may also be valuable to consider risks associated with other cancers in females during pregnancy, such as colorectal cancer, and their potential impact on maternal and fetal outcomes [16].

### Limitations of our study

This was a retrospective study and was conducted at a single center, which may restrict the generalizability of the results. The sample size was relatively small, and although patients with missing data were excluded, some degree of selection bias cannot be excluded. Diagnostic procedures require further clarification. Our model was predictive, but sensitivity analysis was not performed.

As the management plan guidelines and MRI interpretation slightly differ between centers and radiologists, this may have affected the consistency of findings. These factors should be considered when interpreting the findings. Moreover, previous scoring systems for LN metastasis risk stratification have largely focused on low-risk EC patients and have not been widely validated across different populations. Future large-scale, multicenter prospective studies are recommended to validate and expand upon our results.

Importantly, a major limitation of this study is the high prevalence of LN metastasis (53%), which exceeds typical rates reported in the general EC population (10–20%). This reflects referral bias and our center’s policy of staging surgery in high-risk patients, thereby enriching the sample for nodal disease. Consequently, the model’s predictive performance (AUC 0.927) may be inflated, and external validation in broader, mixed-risk populations is essential before general clinical application.

## Conclusions

Most EC patients are diagnosed at an early stage and may not benefit from routine lymphadenectomy, which can increase morbidity without added value. The multivariate analysis found that CA-125, enlarged para-aortic lymph nodes, and large tumor diameter were the key independent predictors of lymph node metastases. Other factors, such as deep myometrial invasion and cervical stromal invasion, showed significant associations in univariate analysis. Routine preoperative assessment of these parameters can help clinicians identify patients who are most likely to benefit from lymphadenectomy or adjuvant therapy, while reducing unnecessary procedures and their associated complications; however, external validation in larger, multicenter prospective studies is required before clinical adoption.

### Recommendations

We recommend that future research should include a comparative analysis between menopausal and non-menopausal patients to better understand potential differences in outcomes. Additionally, separate subgroup analyses for low-grade and high-grade endometrial carcinoma (LGEC and HGEC) are encouraged to provide more specific and clinically relevant insights. Given the novelty of our topic and the limited number of available studies, larger multicenter studies are essential to validate and expand upon our results, as well as to perform sensitivity analyses in future predictive models. A more detailed evaluation of patients using detailed diagnostic parameters is also recommended.

To further strengthen and validate our findings, future studies would benefit from deeper contextualization with established prediction models, such as the Kanagawa Cancer Center and Mayo scoring systems, highlighting both the overlapping and the novel aspects of the current approach.

## Data Availability

The dataset generated and/or analyzed during the current study is available from the corresponding author upon reasonable request.

## Abbreviations

EC: Endometrial Cancer
CA125: Cancer Antigen 125
U/mL: Units per milliliter
AUC: Area Under the Curve
LGEC: low-grade endometrial carcinoma
HGEC: high-grade endometrial carcinoma
LVI: lympho-vascular invasion
